# Dental Materia Medica

**Published:** 1866-05

**Authors:** Geo. Watt


### THE DENTAL REGISTER.

Vol. XX.] MAY 1866. [No. 5.

### Original Communications.

### DENTAL MATERIA MEDICA.

By Geo. Watt.

#### Chlorine, Cl = 35.

Chlorine, when free, is a yellowish green gas. It is one of the elements, and is characterized by strong affinities, and consequently, active properties. It is, accordingly, found in both kingdoms of nature.

Preparation.—It is conveniently obtained by heating hydrochloric acid and binoxyd of manganese in a retort, and collecting the gas as it escapes. As neither ingredient is costly, it is not necessary to be particular as to the relative. quantities. Equal weights of the oxyd and acid will answer. The chemist will understand the reactions of the process by an equation :

$$2HCl + MnO_2 = MnCl + 2HO + CL.$$

To collect the gas pure, the process of displacement is best. Extend the disengaging tube to the bottom of a dry bottle, the air will be driven out as the gas accumulates ; and, by the color of the chlorine, it may be readily seen when the bottle is full, But as the dentist usually wants only its watery solution, the discharging tube may pass into pure water which dissolves a quantity of the gas.

Chlorine is also prepared, and with less expense, by heating in a retort, a mixture of common salt, binoxyd of manganese, and dilute sulphuric acid. Equal weights of salt, manganese, acid, and water will give satisfactory results. The reactions are represented thus:

$$NaCl+MnO_2+2SO_3=NaO,SO_3+MnO,SO_3+Cl.$$

This mixture gives off chlorine freely, with merely the heat of a summer day. Hence it is convenient, as well as cheap in fumigating or disinfecting sinks, etc.

PROPERTIES.—Chlorine is ordinarily gaseous, is two and a half times as heavy as air, has an astringent taste, and a very suffocating odor. It is not combustible, but supports combustion. In the presence of water it destroys vegetable colors, and most offensive odors.

USES.—Chlorine is, probably, the most reliable bleaching and fumigating agent known to science. A dilute solution of chlorine in water is a good mouth wash when there is a tendency to putridity, or where their is an offensive breath. It may also be injected into the cavity of the antrum, to arrest offensive discharges, and to relieve subacute inflammation of its living membrane. In bleaching teeth, it is very reliable; but I prefer the hypochlorite of soda for this purpose, for reasons which will hereafter appear.

MODUS OPERANDI.—How does chlorine bleach and disinfect? That is the question. Mainly, if not wholly by its affinity for hydrogen. Dry chlorine will scarcely, if at all bleach dry tissue. It takes hydrogen from almost anything. When it takes it from water, oxygen is set free, and with its strong affinities, increased by the nascent state, it, too, goes to bleaching. Most coloring matters contain hydrogen, which will most certainly be yielded, when demanded by two such agents as oxygen and chlorine. By taking one element away, the chemical characteristics of the coloring matter are lost; and the remaining elements are left to seek new affinities.

The action is the same in fumigation. Sulphuretted hydrogen, ammonia, and phosphuretted hydrogen, are the ordinary stinks resulting from the putrefaction of organic matter. Chlorine exactly meets the demands of society by taking their hydrogen.

### Hypochlorite of Soda.

This salt is sometimes called "liquid chloride of soda"; but chlorine can not unite directly with soda, hence the name conveys a false impression. It is called "Liquor Sodæ Chlorinatæ" in the United States Dispensatory, and is often sold as "Labarraque's Solution," from the fact that, about 1820, Labarraque discovered its disinfecting powers. But I prefer the name which, while it answers all other purposes, convey a knowledge of its chemical composition, that is, the composition of the compound to which the solution owes its medical properties.

Preparation.—It is most conveniently prepared by action on a solution of carbonate of soda with a solution of hypochlorite of lime, and filtering through a linen cloth, to remove the precipitated carbonate of lime. For full particulars, the reader is referred to the U. S. Dispensatory. The solution is so generally kept by druggists, that we are not often called on to prepare it.

Modus Operandi.—The action of this medicine will be better understood by a careful notice of its composition, and the characteristics of the elements composing it. One equivalent of chlorine, combined with one of oxygen, forms hypochlorous acid. This unites with soda, to form the hypochlorite under consideration. Oxygen and chlorine are held together by a feeble affinity, and are, therefore, easily separated. That is the reason this solution undergoes decomposition in the air, or in contact with organic matter. When decomposed, oxygen, chlorine, and soda are all set free. The soda simply acts as an alkali. The chlorine and oxygen have strong affinities for many other elements, and especially for hydrogen. And these affinities have now the special energy of the nascent state. When free chlorine bleaches in the presence of water, the oxygen only is nascent; but with the hypochlorite both elements have this advantage. Accordingly, we discover by analogy, what is demonstrated by experiment, that the hypochlorite is a better bleacher and disinfectant than chlorine itself. In fumigating and bleaching, the base, soda, does not play an active part. It simply holds the other elements together till they are conveniently brought in contact with the substances which they are to decompose.

USES.—These will be suggested to the dentist, to a great extent, by what has been already said. The bleaching, disinfecting, and antiseptic properties of the hypochlorite, render it peculiarly useful in dental therapeutics. Whenever there is an offensive odor arising from decayed teeth, diseased antrum, or morbid secretions of the mouth, its use is indicated. The odor is at once destroyed by bringing the medicine in contact with the part from which it emanates, It also acts on mucous membranes as a gentle irritant, arousing the vital actions so as to overcome morbid conditions. It is an admirable injection for the antrum, in chronic inflammation of its lining membrane, especially when there is a fetid discharge. When the breath is offensive, the mouth should be washed, and the throat gargled with it; and a small quantity of it may be swallowed with advantage. But with a knowledge of its mode of action, every one will know when to use it. It is *the bleacher.*

ADMINISTRATION.—As a mouth wash and gargle, the solution should be diluted with two or three parts of water. When used in the antrum, a dilute solution frequently applied, till the discharge ceases to be offensive, is preferable to those more concentrated. It may be injected, with profit, into alveolar abscesses, and pulp cavities. That it is *disinfectant* and *antiseptic* cannot be to strongly kept in mind.

In bleaching teeth it should be applied *frequently* two or three times in an hour, when practicable.

### Hypochlorite of Lime.

This salt is called, in commerce and arts, "chloride of lime," in medicine, " chlorinated lime." It is so abundant, and therefore, so readily obtained, that a description of its preparation is not necessary. It is the cheapest and most convenient substance for fumigating sinks, spittoons, dwellings, etc. Its solution in water may be used as a substitute for the " Liquor Sodæ Chlorinatæ," or hypochlorite of soda. Its modus operandi is the same. By adding sulphuric, nitric, acetic or kindred acids to it, it gives off chlorine more rapidly.

